# CGAT: computational genomics analysis toolkit

**DOI:** 10.1093/bioinformatics/btt756

**Published:** 2014-01-05

**Authors:** David Sims, Nicholas E. Ilott, Stephen N. Sansom, Ian M. Sudbery, Jethro S. Johnson, Katherine A. Fawcett, Antonio J. Berlanga-Taylor, Sebastian Luna-Valero, Chris P. Ponting, Andreas Heger

**Affiliations:** CGAT, MRC Functional Genomics Unit, Department of Physiology, Anatomy and Genetics, Parks Road, Oxford OX1 3PT, UK

## Abstract

**Summary:** Computational genomics seeks to draw biological inferences from genomic datasets, often by integrating and contextualizing next-generation sequencing data. CGAT provides an extensive suite of tools designed to assist in the analysis of genome scale data from a range of standard file formats. The toolkit enables filtering, comparison, conversion, summarization and annotation of genomic intervals, gene sets and sequences. The tools can both be run from the Unix command line and installed into visual workflow builders, such as Galaxy.

**Availability:** The toolkit is freely available from http://github.com/CGATOxford/cgat

**Contact:** andreas.heger@dpag.ox.ac.uk

## 1 INTRODUCTION

A central task in computational genomics is to extract biologically meaningful summaries and annotations from short read sequences to facilitate both visualization and statistical analysis. Commonly, this process starts by mapping next-generation sequencing (NGS) reads and quantitating their distribution in genomic features such as transcripts with expression level and transcription factor binding sites with peak scores. This initial contextualization phase is well supported by specialized tools such as Tophat/Cufflinks (Trapnell *et al.*, 2012) or MACS (Feng *et al.*, 2012). In a second phase, datasets are typically integrated to allow interpretation, asking, for example, how many transcription factor binding sites are associated with each of exonic, intronic, flanking and intergenic genomic annotations. This phase necessarily relies on computational tools that can describe, integrate and summarize a variety of feature files produced from the initial phase and external annotation sources. Here we introduce a collection of tools that assist genomic scientists in successfully performing this crucial data integration and interpretation phase, bridging the gap from raw data to biologically interpretable results. We have made extensive use of these tools in a number of NGS projects (Long *et al.*, 2013, Rajan *et al.*, 2013, Ramagopalan *et al.*, 2010).

## 2 OVERVIEW

The computational genomic analysis toolkit comprises >50 tools, each with documentation and examples. Tools are tagged to facilitate discovery. Tags associate tools with broad themes (genomic intervals, gene sets, sequences), standard genomic file formats (BED, GTF, BAM, FASTA/Q) and the type of computation performed by the tool, such as statistical summary, format conversion, annotation, comparison or filtering.

**Fig. 1.**
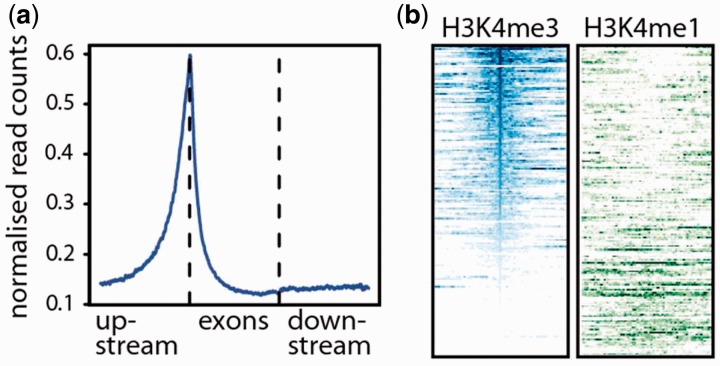
Visualization of the output of (**a**) bam2geneprofile and (**b**) bam2peakshape

As an illustrative example, a gene set can be annotated with the tool *gtf2table.* In fact, *gtf2table* provides >25 different methods to annotate transcript models. Annotation is dependent on auxiliary data: given a genome sequence, transcripts can be annotated by composition (e.g. %GC); given a reference gene set, transcripts can be marked as fragments or extensions, enabling the user to ascertain the completeness of transcript models built for RNAseq data. Given a BAM file with NGS read data, *gtf2table* can compute coverage in sense/antisense direction over transcript models; another example, *bam2geneprofile* computes and plots metagene-profiles from mapped NGS read data in BAM format (Fig. 1a). Different metagene models (with/without UTRs/introns, etc.) and various normalization options are available. Finally, the tool *bam2peakshape* computes read densities in specified genomic intervals to generate matrix data suitable for visualization in heatmaps (Fig. 1b). The toolkit also contains standard sequence analysis utilities such as *fasta2table*, which annotates sequences with CpG frequencies, codon frequencies and amino acid composition. To assist the interpretation of NGS data, the toolkit implements various classification schemes for transcript data or interval data. RNA-seq-derived transcripts can be marked as instances, fragments, extensions or alternative versions of transcripts in a reference gene set. Chromatin immunoprecipitation-sequencing (ChIP-Seq) intervals can be marked as intronic, intergenic or within the UTR, upstream or downstream regions of transcript models. Finally, the toolkit provides tools to summarize genomic datasets, reporting the number of intervals or transcripts per chromosome, size distributions of features and more.

## 3 USAGE

We introduce the usage of the computational genomics analysis toolkit with a brief example. The fully worked example can be found online. Given a set of transcription factor binding intervals from a ChIP-seq experiment in BED format (*nfkb.bed*), we wish to determine how many binding intervals lie within exons, introns or intergenic sequence using a reference gene set from GENCODE (Harrow *et al.*, 2012), in GTF format (*hg19.gtf*). We then want to plot the density of binding relative to transcript models and examine the chromatin signatures within the intervals:

cgat **gtf2gtf–**sort = gene < hg19.gtf
| cgat **gtf2gtf–**merge-exons-with-utr^(1)^
| cgat **gtf2gtf**–filter = longest-gene^(2)^
| cgat **gtf2gff–**flank=5000–method = genome^(3) ^> *annotations.gff*
cgat **bed2table–**filename-gff = *annotations.gff*
 –counter=classifier-chipseq<nfkb.bed>*annotated_peaks.tsv*^(4)^

The above sequence of Unix commands in turn (1) merges all exons of alternative transcripts per gene, (2) retains the longest gene in the case of overlapping genes and (3) annotates exonic, intronic, intergenic and flanking regions (size = 5 kb) within and between genes. Choosing different options can provide different sets of answers. Instead of merging all exons per gene, the longest transcript might be selected by replacing (2) with *gtf2gtf – filter = longest-transcript*. Note that the creation of *annotations.gff`* goes beyond simple interval intersection, as gene structures are normalized from multiple possible alternative transcripts to a single transcript that is chosen by the user depending on what is most relevant for the analysis.

The generated annotations are then used to classify the transcription factor binding sites using *bed2table* (4). The profile of ChIP-seq binding over genes can be calculated and plotted using *bam2geneprofile* (Fig. 1a). Chromatin state at ChIP-seq peaks can be investigated by integrating H3K4me1 and H3K4me3 data for a relevant tissue (ENCODE Project Consortium *et al.*, 2012) using *bam2peakshape* and plotted in *R* (R Core Team, 2012) (Fig. 1b). Statistical significance can be assessed using tools such as GAT (Heger *et al.*, 2013). More usage examples, including testing for functional enrichment, assessment of CpG content in long non-coding RNA promoters and clustering metagenomic contigs on tetranucleotide frequency, can be found online.

## 4 IMPLEMENTATION

We aim to write legible and maintainable code that can serve as an entry point into computational methods for biologists. The toolkit is implemented in the Python language (van Rossum, 1995). Some performance-critical sections have been implemented in Cython (Behnel *et al.*, 2011). The toolkit can be installed from common Python package repositories. Dependencies will be installed automatically, although some tools require external software to be installed. All tools are freely available under the BSD 3-clause licence. The toolkit is under constant development, and community involvement in the project is welcome. Regression tests ensure that core functionality is maintained as scripts are extended. All tools are built using a common coding style and follow a naming scheme centred on common genomic file formats. The tools have a consistent command line interface enabling them to be combined into work flows using Unix pipes and integrated into automated pipelines allowing automated and parallel execution. They use a consistent logging mechanism to facilitate issue tracking. Furthermore, the use of common genomic formats means that tools can be easily combined with other popular genomic software such as BEDtools (Quinlan and Hall, 2010), University of California, Santa Cruz tools (Kuhn *et al.*, 2013) or biopieces, http://www.biopieces.org. An RDF (Resource Description Framework, http://www.w3.org/RDF) description of each tool can be generated for use with tools such as CLI-MATE (Tatum *et al.*, 2011) to generate XML files for a variety of workflow frameworks, such as Galaxy (Goecks *et al.*, 2010).

*Funding*: This work was funded by the UK Medical Research Council.

*Conflict of Interest*: none declared.
